# Current and Future Disease Progression of the Chronic HCV Population in the United States

**DOI:** 10.1371/journal.pone.0063959

**Published:** 2013-05-21

**Authors:** Martin Zalesak, Kevin Francis, Alex Gedeon, John Gillis, Kyle Hvidsten, Phyllis Kidder, Hong Li, Derek Martyn, Leslie Orne, Amanda Smith, Ann Kwong

**Affiliations:** 1 Trinity Partners, LLC, Waltham, Massachusetts, United States of America; 2 Vertex Pharmaceuticals Incorporated, Cambridge, Massachusetts, United States of America; Centers for Disease Control and Prevention, United States of America

## Abstract

Chronic hepatitis C virus (HCV) infection can lead to advanced liver disease (AdvLD), including cirrhosis, decompensated cirrhosis, and liver cancer. The aim of this study was to determine recent historical rates of HCV patient progression to AdvLD and to project AdvLD prevalence through 2015. We first determined total 2008 US chronic HCV prevalence from the National Health and Nutrition Evaluation Surveys. Next, we examined disease progression and associated non-pharmacological costs of diagnosed chronic HCV-infected patients between 2007–2009 in the IMS LifeLink and CMS Medicare claims databases. A projection model was developed to estimate AdvLD population growth through 2015 in patients diagnosed and undiagnosed as of 2008, using the 2007–2009 progression rates to generate a “worst case” projection of the HCV-related AdvLD population (i.e., scenario where HCV treatment is the same in the forecasted period as it was before 2009). We found that the total diagnosed chronic HCV population grew from 983,000 to 1.19 million in 2007–2009, with patients born from 1945–1964 accounting for 75.0% of all patients, 83.7% of AdvLD patients, and 79.2% of costs in 2009, indicating that HCV is primarily a disease of the “baby boomer” population. Non-pharmacological costs grew from $7.22 billion to $8.63 billion, with the majority of growth derived from the 60,000 new patients that developed AdvLD in 2007–2009, 91.5% of whom were born between 1945 and 1964. The projection model estimated the total AdvLD population would grow from 195,000 in 2008 to 601,000 in 2015, with 73.5% of new AdvLD cases from patients undiagnosed as of 2008. AdvLD prevalence in patients diagnosed as of 2008 was projected to grow 6.5% annually to 303,000 patients in 2015. These findings suggest that strategies to diagnose and treat HCV-infected patients are urgently needed to increase the likelihood that progression is interrupted, particularly for patients born from 1945–1964.

## Introduction

While some patients initially infected with hepatitis C virus (HCV) spontaneously clear the infection and become HCV RNA negative, the majority (∼70%) of patients become chronically infected [1], [2], [3]. The incidence of new HCV infections has recently been low and declining [4], with the implementation of routine blood screening decreasing transfusion-related infection [5]. As a result, intravenous drug use is now the primary cause of transmission, with sexual transmission playing a lesser role [5], [6]. Historical incidence trends point towards a wave of HCV infections occurring in individuals born between 1945 and 1964 (i.e., the “baby boomer” population), [3], [7] resulting in a total United States (US) prevalence estimated to approach 4 million patients, [8], [9] with many infected individuals having lived with latent disease for decades. The US prevalent population may surpass 5 million if homeless, institutionalized, and military patients are included [10]. HCV has been associated with increased mortality regardless of the extent of liver disease [9], [11], and deaths from HCV now surpass HIV-associated mortality in the US [12]. The longer a patient is chronically infected with HCV, the higher the chance of progression to advanced liver disease (AdvLD), namely cirrhosis, decompensated cirrhosis, and liver cancer [2]. Approximately 20% of chronically infected patients develop cirrhosis within 20 to 30 years of the initial infection [3], [13], [14], [15], although there is some variability across specific subpopulations, with certain co-factors (e.g., age, HCV genotype 3 infection, alcohol abuse, diabetes) contributing to more rapid progression [16], [17], [18], [19], [20], [21], [22]. Patients who progress to decompensated cirrhosis may present with potentially life-threatening complications (e.g., hepatic encephalopathy) [15], and become candidates for liver transplantation. For liver transplantation in the US, HCV is implicated in two ways: i) it is the leading indication for liver transplantation in the US (39% in 2006), [23] and ii) it is one of the leading causes of liver cancer, another key indicator for transplant (23%) [24].

The medical burden associated with HCV translates into a substantial cost burden to the US healthcare system as well, although there are few recent studies. In one historical study, total cost related to HCV infection in the US was estimated at $5.5 billion in 1997, with about a third attributed to direct medical costs [25]. Another study estimated the total direct healthcare cost at more than $1 billion for 1998 [8]. A more recent analysis found that all-cause per HCV-infected patient costs have been rising and the annual all-cause costs per patient ($20,961) between 2002–2006 were nearly four-fold greater than that of matched controls ($5,451) [26]. In view of the high and growing morbidity and costs associated with HCV, several studies have recently focused on demonstrating how increased diagnosis and new treatment modalities could have a positive influence on patient- and cost-related outcomes [27], [28], [29], [30], e.g., the initiation of a birth cohort-focused screening HCV program that complements a behavioral risk-driven approach would be highly cost-effective and would substantially increase chronic HCV diagnosis rates.

The aim of this study was to determine the recent burden of HCV, especially HCV-associated AdvLD, in the US using adjudicated claims data, and project it forward through 2015. We began by estimating the US prevalence of chronic HCV in 2008. Next, we used historical medical claims data from 2007–2009 to determine the prevalence and the non-pharmacological treatment costs of civilian, non-institutionalized non-AdvLD and AdvLD patients in this time period. We then projected AdvLD prevalence in the US chronic HCV population through 2015 based on progression and mortality rates observed in the claims data. Our dynamic progression model represents the “worst case” scenario where historical screening/diagnosis rates and no treatment beyond therapies available prior to 2009 is applied. The model uses progression rates derived directly from 2007–2009 claims data of HCV-infected patients who may or may not have been treated with antiviral therapy available at that point of time. Thus, it represents the scenario where diagnosis and treatment of HCV-infected patients is the same in the forecasted period through 2015 as it was prior to 2009. This “worst case” scenario portrays the situation in the absence of direct-acting antivirals and/or new screening programs that use other co-factors other than those currently used to identify at-risk patients (e.g., intravenous drug users). The intent is to highlight the urgency of identifying currently undiagnosed patients and effectively treating both currently diagnosed and undiagnosed patients with state-of-the-art antiviral treatment, as lack of treatment can result in a large number of patients progressing to AdvLD and incurring significant healthcare costs during the foreseeable period.

Although previous studies have estimated the future prevalence and cost of chronic HCV infection in the US, [31], [32], [33], [34], [35], [36], this is the first study to derive HCV-related AdvLD prevalence, disease progression, and mortality rates using recent adjudicated claims data.

## Materials and Methods

### Estimation of US HCV Population

The total US HCV prevalence, and the viremic (i.e., chronic) HCV prevalence were estimated using data from the 2005–06 and 2007–08 National Health and Nutrition Evaluation Survey (NHANES) [37], in which a total of 19,712 survey participants were sampled during the two iterations used in this study. The NHANES dataset is a publicly available resource that can be downloaded without cost from the CDC website. HCV prevalence was determined by a positive test for HCV antibody, and the prevalence of chronic HCV infection was determined by a positive HCV RNA test result. The HCV antibody and RNA test data were obtained from the 2005–06 and 2007–08 NHANES survey Laboratory files. Using data from the 2005–06 and 2007–08 NHANES survey Questionnaire files, chronic HCV-infected patients were further categorized into undiagnosed and diagnosed based on their awareness of a previous diagnosis.

### Analysis of Claims Data

To estimate the number of chronic HCV-infected patients in the US Medicare system and their stage of disease, data were obtained from the 2007–2009 CMS Medicare claims database and the LifeLink claims database (IMS LifeLink: Health Plan Claims Database, all rights reserved). The CMS claims data is a publicly available limited data set that can be downloaded from the CMS website, and covers the US population aged 65 and over, patients of all ages who are disabled, those with end-stage renal disease (ESRD), and those who are on Social Security or receiving Railroad retirement benefits [38]. It comprises 100% of the 5-year Medicare claims data for both outpatient and inpatient institutional settings and 5% of the carrier data from the physician office setting, which was projected to national equivalents. The LifeLink claims database (acquired from IMS Health Incorporated) includes patients <65 years old that were commercially or self-insured, receiving assistance from a State Children’s Health Insurance Program (SCHIP), commercially sponsored Medicaid plans, and commercially sponsored Medicare plans not included in the Medicare data.

The data was analyzed using SQL Server to create a relational database that utilized a de-identified patient number as the common identifier. Patients with HCV were defined as having an HCV-related ICD-9-CM diagnosis code on a claim in any given year. HCV patient claims were further analyzed for AdvLD using ICD-9-CM diagnosis codes (see Table S1 for the ICD-9-CM codes used). HCV patient claims were divided into five-year birth cohorts except for those born after 1984, who were excluded from the 5-year cohort analysis due to small sample size. Annual costs for a patient were determined only if the patient had an HCV-related claim in that year. If so, then reimbursed amounts were totaled for all claims where ICD-9-CM codes for HCV and/or AdvLD were indicated as either the primary or secondary diagnosis. LifeLink patient and reimbursed cost data were indexed to 2007 to control for differences in LifeLink enrollment numbers each year. All numbers from the LifeLink analysis were projected to the non-Medicare diagnosed chronic HCV population total.

### HCV Patient Disease Progression Rates (2008–2009)

IMS LifeLink claims data from 2007–2009 were used to calculate disease progression rates by conducting a retrospective cohort study that analyzed HCV-diagnosed patients with or without AdvLD. Patients were followed for one year (from 2008 to 2009) to determine whether they progressed to a different disease state. The rates were derived from the percentage of patients with an HCV-related disease state in 2008 that progressed to another HCV-related disease state in 2009 based on the patient progression rules shown in Figure S1, whereby patients only progress to more severe complications, with the exception of patients with decompensated cirrhosis reverting back to cirrhosis. To ensure an adequate number of events were present in the data to enable accurate calculation of progression rates to less common AdvLD states, patients were divided into three age cohorts (<45 years old, 45–64 years old, and 65+ years old). Patient status in 2007 was used to determine whether they were newly diagnosed or existing patients, and also helped define the patient’s status in 2008. All patients who were undiagnosed as of 2008 were assumed to be non-AdvLD patients.

### HCV Patient All-Cause Mortality Rates (2008)

Medicare claims data from 2008 were used to determine mortality rates for each HCV-related disease state, divided into 5-year age cohorts, by calculating the percentage of patients that died during the calendar year (Figure S2). As such, these mortality rates only reflect the diagnosed HCV population. The Medicare HCV population mortality rate was calibrated to that of the general population by dividing the total US mortality rate (obtained from 2007 US Census Bureau data [39]) by the total Medicare mortality rate; this factor was used to determine the mortality rates to be used in the progression model described below.

### Projection of AdvLD Progression (2008–2015)

A dynamic patient progression modeling framework was constructed using Microsoft Excel® (Figure S3). The model predicted the evolution of the diagnosed and undiagnosed HCV-infected population from 2008 onwards based on the age-matched progression and mortality rates outlined above. All chronic HCV-infected patients who were undiagnosed as of 2008 were assumed to be non-AdvLD as a conservative estimate. Liver transplants were kept constant at 2,400 per year. The model also assumed that undiagnosed patients had the same age distribution as the diagnosed population in 2008, which was validated by an analysis of the NHANES data used to estimate the US HCV population (Figure S4).

### Projection Model Sensitivity Analysis

The sensitivity of the projection model described above to the starting number and distribution of non-AdvLD/AdvLD patients was tested by replacing the 2008 diagnosed HCV population with the analogous values determined for the non-AdvLD/AdvLD population distributions in 2007 or 2009 (see Table S2). Concomitantly, the 2008 undiagnosed HCV population was replaced with calculated values for undiagnosed HCV patients in 2007 or 2009, which were derived from the 2008 value by adjusting for annual chronic HCV incidence [40], mortality rates of undiagnosed HCV patients (based on the rates for diagnosed non-AdvLD patients previously described), and the number of diagnosed HCV patients in 2008 compared to 2007 and 2009. The 2015 AdvLD population derived from replacement of the 2008 HCV population with 2007 or 2009 was compared the original value based on 2008 to ascertain the validity of our model projections and statistical analyses.

## Results

### Estimated 2008 US HCV Population

Of the 2008 US Census 16–85 year old population of 232.9 million [41], 3.57 million (a total prevalence of 1.53%) were estimated to have been infected with HCV at some point based on the 2005–06 and 2007–08 NHANES studies (Figure S5) [37]. An estimated 2.68 million (75.1%) of the population had chronic HCV in 2008 (i.e., were viremic). This group was further segmented into 1.10 million diagnosed patients, and the remaining 1.58 million (59%) who were undiagnosed at the time of the NHANES study.

### Overall US HCV Population and Cost Dynamics

The estimated diagnosed US HCV population grew from 983,000 to 1.19 million in 2007–2009 (annual growth rate of 10.1%; see Figure S6). AdvLD population growth totaled 60,000 new patients (annual rate of 16.6%), outpacing that of the non-AdvLD population (8.7%) in the two-year period. By 2009, AdvLD patients accounted for 19.1% of the diagnosed HCV population, compared to 17.0% in 2007. Decompensated cirrhosis and cirrhosis were the most common forms of AdvLD, comprising 46.6% and 36.5% of the 2009 AdvLD HCV population, respectively, and contributed the majority of the 60,000 new AdvLD patients in 2009 (30,000 and 18,000 new patients, respectively).

In a similar manner to the growth in the HCV population, the overall non-pharmacological cost burden of HCV-infected patients grew from $7.22 billion in 2007 to $8.63 billion in 2009 at an annual growth rate of 9.4% (Figure S6), a substantially higher growth rate than for total US healthcare costs over the same period (4.3%) [42]. Analogous to patient growth, cost growth was higher in the AdvLD cohort (13.2%) compared to non-AdvLD (4.0%).

**Figure 1:**
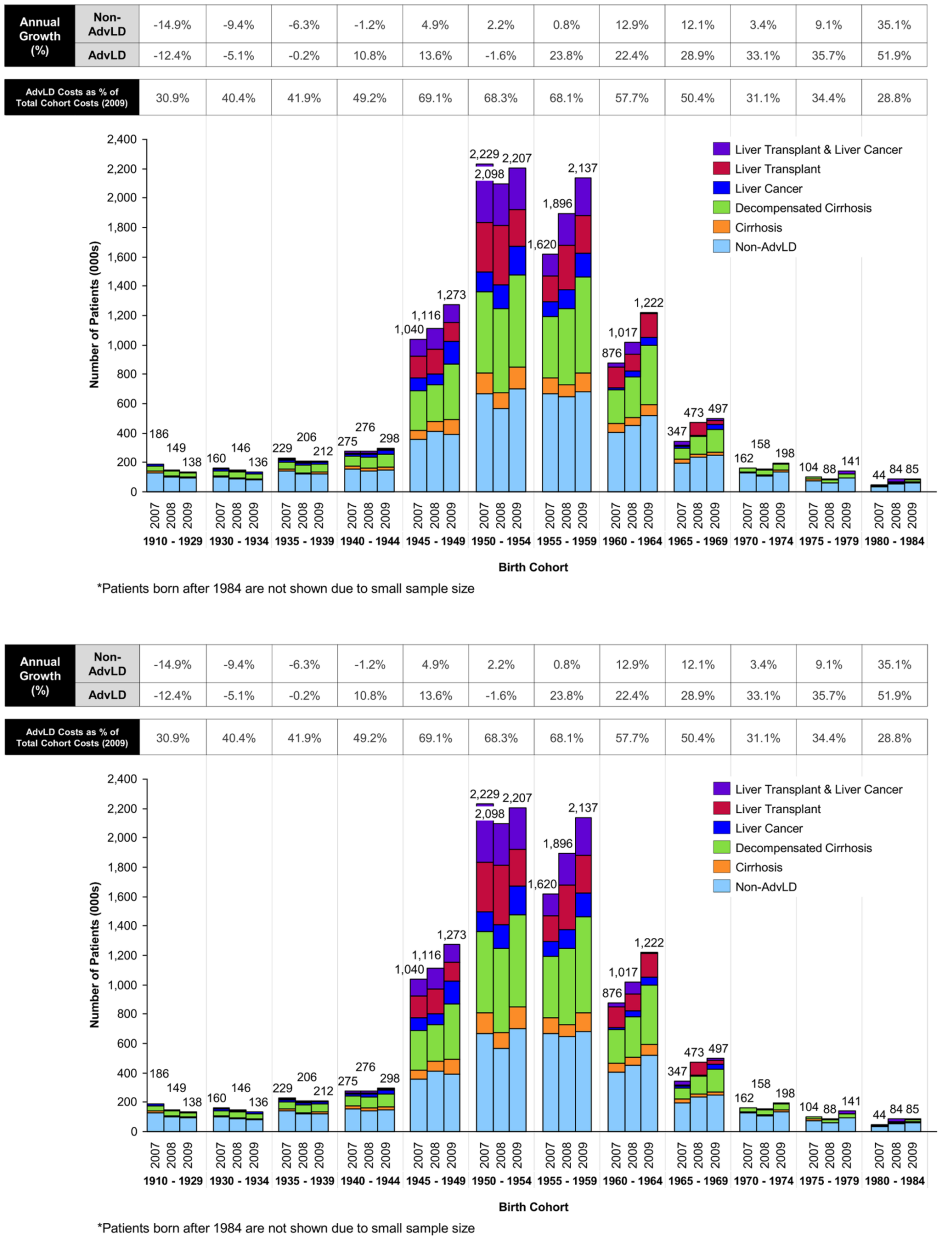
Total non-AdvLD/AdvLD patients and costs and their 2007–2009 growth. (A) Total non-AdvLD/AdvLD patients and their 2007–2009 growth rates by birth cohort. (B) Total non-AdvLD/AdvLD costs and their 2007–2009 growth rates by birth cohort.

The birth cohort analysis revealed that patients born in 1945–64 were the largest age group among the different age groups and that they are rapidly progressing to AdvLD. This cohort accounted for 75.0% of all HCV-infected patients in 2009 (Figure 1A), and in the AdvLD population the concentration of patients born in 1945–64 is even more striking, with 83.7% of patients belonging to this age group. This cohort experienced particularly rapid progression to advanced disease, highlighted by the 1955–59 group, where the AdvLD population increased by 22.9% annually (see Table S3 for growth rates by disease state for each cohort). The 1945–64 cohort also contributed the vast majority (91.5%) of new AdvLD patients.

Analysis of cost data by birth cohort revealed that the 1945–1964 birth cohort accounted for 79.2% of total and 85.9% of AdvLD-related costs in 2009. All groups except for the three oldest cohorts experienced increased costs in 2009 compared to 2007 (Figure 1B). Generally, the growth in AdvLD costs exceeded that of the non-AdvLD group (see top two rows of table in Figure 1B). The contribution of AdvLD patients to overall cost increases peaked amongst the four middle-aged cohorts. Among AdvLD categories, decompensated cirrhosis contributed the greatest proportion of total cost (29.2%) in 2009, despite representing only 8.9% of patients (Table 1).

**Table 1:** Percentage contribution of each HCV disease state to 2009 total patients and costs.

	Non-AdvLD	Cirrhosis	Decomp. Cirrhosis*	Liver Cancer	Liver Transplant	Liver C/T**
**% Contribution to Total Patients**	80.9%	7.0%	8.9%	1.5%	1.3%	0.4%
**% Contribution to Total Costs**	38.7%	6.1%	29.2%	7.8%	10.0%	8.2%

* Decomp. = decompensated,

** C/T = cancer/transplant.

In contrast to the other groups, the three oldest birth cohorts experienced decreases in the number of patients and totals costs between 2007–09 (see Figures 1A and 1B), likely as a result of the increased all-cause mortality rate in this older population (see Figure S2). Across these three cohorts, the total HCV population decreased by 11,000 between 2007–09 (annual rate of −9.3%), with a concomitant drop in HCV-related non-pharmacological treatment costs from $576 million in 2007 to $503 million in 2009 (annual rate of −6.5%). Separating into different disease status, AdvLD and non-AdvLD patient populations decreased at similar annual rates (−9.5% and −9.3% respectively), however there was a more substantial annual cost decrease in the non-AdvLD group (−10.1%) compared to the AdvLD cohort (−4.8%).

### Disease Progression Rates in the HCV Patient Population

For all patients, disease progression rates in 2008–2009 between the various AdvLD states were much higher than for patients progressing from non-AdvLD to an AdvLD state (Tables 2, 3, 4). In the <45 year old cohort, 1.6% of non-AdvLD patients progressed to AdvLD annually, which increased to 4.4% and 5.2% in the 45–64 and 65+ year old cohorts respectively. By comparison, progression of HCV-related cirrhosis patients to decompensated cirrhosis alone occurred at rates of 5.1%, 11.5% and 8.8% in the <45, 45–64 and 65+ year old cohorts, respectively. In all three cohorts, both the forward and “reverse” progressions between cirrhosis and decompensated cirrhosis were similar. This is to be expected as decompensation is a potentially reversible complication of cirrhosis.

**Table 2:** Annual progression rates by HCV patient disease status in the <45 y.o. age cohort.

		Resultant Patient Disease State				
		Cirrhosis	Decomp. Cirrhosis*	Liver Cancer	Liver Transplant	Liver Cancer/Transplant
**Original Patient** **Disease State**	**Non-AdvLD**	0.78%	0.68%	0.03%	0.03%	0.03%
	**Cirrhosis**	N/A	5.10%	1.02%	0.03%	0.03%
	**Decomp. Cirrhosis** *	3.25%	N/A	0.65%	1.30%	0.03%
	**Liver Cancer**	N/A	N/A	N/A	N/A	0.03%
	**Liver Transplant**	N/A	N/A	N/A	N/A	0.03%

* Decomp. = decompensated.

**Table 3:** Annual progression rates by HCV patient disease status in the 45–64 y.o. age cohort.

		Resultant Patient Disease State				
		Cirrhosis	Decomp. Cirrhosis*	Liver Cancer	Liver Transplant	Liver Cancer/Transplant
**Original Patient** **Disease State**	**Non-AdvLD**	2.23%	1.81%	0.25%	0.05%	0.01%
	**Cirrhosis**	N/A	11.45%	2.37%	0.49%	0.33%
	**Decomp. Cirrhosis** *	11.56%	N/A	1.38%	2.33%	0.45%
	**Liver Cancer**	N/A	N/A	N/A	N/A	9.16%
	**Liver Transplant**	N/A	N/A	N/A	N/A	5.84%

* Decomp. = decompensated.

**Table 4:** Annual progression rates by HCV patient disease status in the 65+ y.o. age cohort.

		Resultant Patient Disease State				
		Cirrhosis	Decomp. Cirrhosis*	Liver Cancer	Liver Transplant	Liver Cancer/Transplant
**Original Patient** **Disease State**	**Non-AdvLD**	2.51%	2.31%	0.34%	0.00%	0.00%
	**Cirrhosis**	N/A	8.75%	3.13%	0.00%	0.00%
	**Decomp. Cirrhosis** *	7.37%	N/A	2.15%	2.30%	0.00%
	**Liver Cancer**	N/A	N/A	N/A	N/A	5.41%
	**Liver Transplant**	N/A	N/A	N/A	N/A	4.76%

* Decomp. = decompensated.

### All-Cause Mortality Rates in the HCV Patient Population

Two key trends emerged from the analysis of mortality rates in the Medicare 2008 HCV population. First, annual all-cause mortality increased with patient age across all HCV-related disease states (Figure S2). Second, mortality rates increased as the severity of AdvLD increased, with decompensated cirrhosis and liver cancer having the highest annual all-cause mortality rates, exceeding >20% for patients aged 74+ years old. Patients with cirrhosis, the mildest form of AdvLD, have mortality rates higher than those of non-AdvLD patients across all age cohorts. For patients born before 1945, annual all-cause mortality outpaced disease progression, resulting in a lower overall proportion of AdvLD patients in this cohort (see bottom row of table in Figure 1A).

### Projected AdvLD Prevalence through 2015 in HCV-infected Patients Diagnosed and Undiagnosed as of 2008

**Figure 2:**
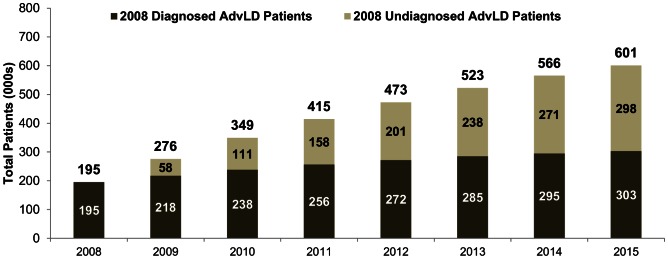
Projected 2008–2015 AdvLD prevalence in HCV-infected patients who were diagnosed and undiagnosed as of 2008, respectively.

**Figure 3:**
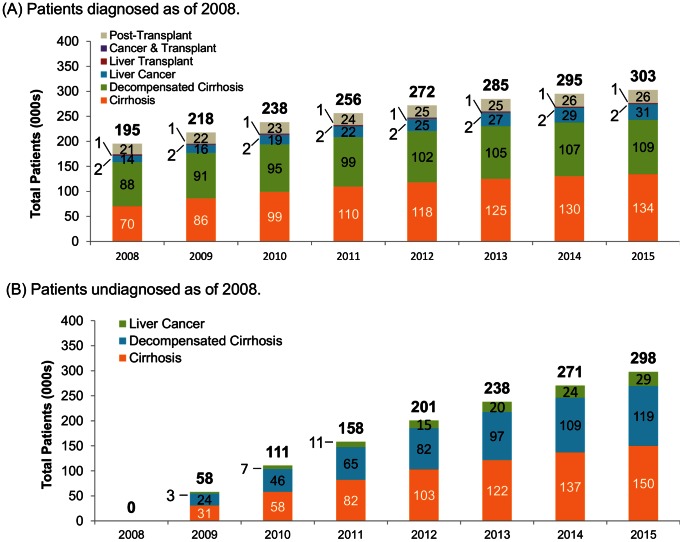
Projected 2008–2015 populations for each AdvLD state in HCV-infected patients. (A) Patients diagnosed as of 2008. (B) Patients undiagnosed as of 2008.

In the absence of potential increases in disease screening, treatment or treatment success rates, the overall AdvLD patient population was projected to grow from 195,000 to 601,000 between 2008 and 2015, largely as a result of rapid progression of AdvLD among the 2008 undiagnosed HCV-infected population (Figure 2). The majority (73.5%) of the 406,000 new AdvLD patients that are expected to emerge by 2015 originate from the patient population that was undiagnosed as of 2008. Thus, by 2015 the AdvLD patient population is projected to be almost equally derived from HCV-infected patients who were undiagnosed and diagnosed in 2008. While growth was projected to be slower in AdvLD patients who were diagnosed as of 2008, this population will still increase at 6.5% annually to reach 303,000 patients by 2015. Within AdvLD, cirrhosis and decompensated cirrhosis patients are projected to represent the majority of AdvLD patients by 2015, and account for 80.2% and 90.4%, respectively of the AdvLD population among patients who were diagnosed or undiagnosed as of 2008 (Figure 3A–B).

### Projection Model Sensitivity Analysis

The sensitivity ofour projection model to the starting number and non-AdvLD/ALD distribution of patients was tested by replacing the 2008 diagnosed and undiagnosed HCV populations with the equivalent populations determined for 2007 and 2009. Using 2007 patient numbers resulted in a total AdvLD population of 588,000 in 2015, 2% lower than the 601,000 AdvLD patients in the original projection. Conversely, using 2009 patient numbers afforded a total AdvLD population of 616,000 in 2015, 3% higher than the original projection.

## Discussion

This retrospective analysis of real-world US HCV-associated non-pharmacologic 2007–2009 claims data indicates that a large increase in the number of patients with HCV-associated AdvLD, especially those born in 1945–1964 (i.e., “baby boomers”), may significantly impact the US healthcare system in the coming years if HCV-infected patients are left undiagnosed and/or untreated. Specifically, many patients who were undiagnosed as having HCV as of 2008 may present with AdvLD by the year 2015.

First, and in line with previous estimates, our analysis of the 2005–2006 and 2007–2008 NHANES datasets showed HCV prevalence of 3.57 million people in the 16–85-year old US population in 2008, and within this group, 2.68 million who were chronically infected. This estimate of chronic HCV prevalence was similar to one based on 1988–1994 NHANES data (2.7 million patients) [43], and somewhat lower than two previous studies based on the earlier 1999–2002 NHANES data (3.2–3.3 million patients) [32] [44], which may have resulted from the use of different NHANES datasets and patient projections. The proportion of the chronically-infected HCV population with AdvLD determined in this study (17.0% in 2007, increasing to 19.1% in 2009) was analogous to another recent report showing that 20% of diagnosed HCV-infected patients had AdvLD based on claims data from 1998–2006 [45]. Population-based models previously projected the total number of AdvLD cases to reach 800,000 by 2009, of which approximately 20,000 were expected to have decompensated cirrhosis [32]. Our retrospective analysis covers only diagnosed HCV-infected patients and shows a lower total number of AdvLD cases but a greater occurrence of decompensated cirrhosis, exceeding 100,000 cases in 2009.

In parallel with increasing AdvLD prevalence, the HCV-associated non-pharmacological costs increased at an annual rate of 9.4% from 2007–2009, more than doubling the 4.3% overall growth in US healthcare costs in the same period [42]. The cohort born between 1945–1964 was again the primary driver of this growth. Because AdvLD development is a function of time lived with the virus, the cost of treating HCV is likely to grow further as the large 1945–1964 birth cohort continues to age. Importantly and perhaps surprisingly, commercial payers are likely to incur most of these costs, since the majority of the patients in the 1945–1964 birth cohort are mostly covered by commercial insurance and are at least several years away from reaching Medicare eligibility.

Our progression model projected significant growth in the US HCV disease burden over the next several years in the absence of diagnosis and treatment beyond those available prior to 2009. In the model, which used progression and mortality rates determined during a time period when pegylated interferon and ribavirin (Peg-IFN/RBV) was the standard of care (i.e., no protease inhibitors), the total AdvLD population would grow rapidly, from 195,000 to 601,000 between 2008–2015, with 73.5% of the new AdvLD patients undiagnosed as of 2008. In the population diagnosed as of 2008, AdvLD was projected to be prevalent in 33.2% of patients in 2015, compared to 17.8% in 2008. The total population of patients with cirrhosis was projected to grow from 70,000 to 285,000 in 2015, creating a much larger pool of patients that could progress to decompensated cirrhosis after 2015, exposing the healthcare system to further elevation of costs. The HCV-related decompensated cirrhosis population itself is projected to reach 228,000 patients by 2015, which exceeds the ∼125,000 patients estimated in previous studies, [32], [34] suggesting a significantly faster progression of chronic HCV-infected patients to decompensated cirrhosis than was previously anticipated. The model further projected that all-cause mortality would also accelerate because patients with decompensated cirrhosis and liver cancer experience a 3- to 10-fold increase in mortality compared to non-AdvLD patients. These findings reveal the importance of establishing integrated programs that expand current diagnosis programs to a wider population, increase physician referral, and utilize direct-acting antivirals as the standard of care for HCV pharmacological therapy due to their higher efficacy [46] and broader treatment inclusion criteria, so that the morbidity, mortality and costs associated with progression to AdvLD are better controlled.

The use of adjudicated Medicare and commercial medical claims allowed us to determine real-life HCV population dynamics. However, this study faced some limitations inherent to the nature of these datasets. The time-limited and observational nature of the claims data did not allow for ascertainment of causality between HCV infection and the presence of an AdvLD condition. Further, only non-pharmacological costs were included in the cost estimates, which may underestimate the true total cost (i.e., medical and pharmacy costs) associated with chronic HCV and AdvLD complications. Conversely, the inclusion of claims where HCV and/or AdvLD is a secondary diagnosis may have led to an overestimation of total non-pharmacological costs related to the treatment of HCV and AdvLD. In addition, the progression model for HCV disease in this study did not include treatment intervention or any efforts to proactively screen at-risk patients undiagnosed as of 2008. Progression rates in this model accounted for current co-factors related to disease progression at a population-wide level using 2007–2009 rates; co-factors that could potentially emerge after 2009 were not considered. It therefore portrays a “worst case” scenario in the absence of direct-acting antivirals and/or new screening programs compared with those in existence prior to 2009. However, we also took a conservative approach by operationally defining all undiagnosed HCV-infected patients as of 2008 to be non-AdvLD, even though HCV-related cirrhosis can be completely asymptomatic [15], so as to avoid overestimating present and projected AdvLD prevalence. Our projection model, which was based on the diagnosed and undiagnosed HCV populations ascertained from recent historical claims and NHANES data, gave similar projections of the 2015 AdvLD population when the initial model year’s population values was changed from 2008 to 2007 or 2009. This finding supports the validity of our modeling methodology and statistical analysis for the desired estimation of the “worst case” 2015 AdvLD population.

Overall, this analysis supports recent studies pointing to the urgent need for increased efforts to diagnose and treat chronic HCV. Considering the morbidity, mortality and cost associated with AdvLD [8], [11], these patients will present a significant burden to the healthcare system if appropriate steps are not undertaken to identify them for treatment with direct-acting antiviral therapy, which is the current standard of care for HCV treatment. Recent analyses projected that treatment with direct-acting antivirals plus Peg-IFN/RBV is a cost-effective intervention for reducing the lifetime risk of AdvLD in genotype 1 HCV-infected patients and improving quality adjusted life years (QALY) [27]. Even Peg-IFN/RBV treatment alone has demonstrated decreased all-cause mortality in US veteran [47] and Canadian/European tertiary care center [48] populations, or a cost-effective increase in QALY [49], [50]. It is important to note that the treatment-induced SVR in these studies does not necessarily decrease all-cause mortality to that of the general population, as these cohorts (e.g. US veterans) may have other co-morbidities that contribute to a higher all-cause mortality rate as they age.

Our study also highlights the public health concern associated with the low rate of HCV diagnosis in the community. Given that less than half of the 2.7 million chronic HCV-infected patients were diagnosed in 2008, the substantial amount of disease progression seen in the 2007–2009 historical claims data only foreshadows the possibility of a larger future impact of HCV on the US population [32]. Our analysis shows that most patients who may potentially progress to AdvLD by 2015 will be from the group undiagnosed as of 2008. The vast majority of these patients, in turn, will come from the 1945–1964 birth cohort. Because HCV infection can be asymptomatic even in patients with cirrhosis [15], and risk-based screening is ineffective [12], many chronic HCV-infected patients may not become diagnosed until they present with severe AdvLD complications. Screening programs that identify and provide treatment for undiagnosed patients have the potential to lessen total HCV cost burden by decreasing the volume of patients requiring expensive treatment for HCV-related AdvLD and reducing their high mortality rate. The impact of screening programs is supported by recent studies [28], [29], highlighting the cost-effectiveness of HCV screening in the 1945–1964 birth cohort, which is shown here to have the highest potential burden of HCV complications. Indeed, the CDC recently recommended that all individuals born from 1945–1964 (“baby boomers”) receive a one-time test for HCV [51], [52].

## Supporting Information

Figure S1 **Rules for HCV patient disease progression.**
(TIF)

Figure S2 All-cause mortality rates by HCV-infected patient disease status*.
(TIF)

Figure S3 **Progression model flow.**
(TIF)

Figure S4 **Age distribution of diagnosed and undiagnosed HCV-infected patients using 2005–06, 2007–08 NHANES data.**
(TIF)

Figure S5 **Prevalence of HCV in the US in 2008 by infection type (viremic/non-viremic).** The viremic (i.e. chronic) population is further divided by diagnosis status.
(TIF)

Figure S6 Total US diagnosed chronic HCV population (2007–2009): total patient count and total costs by AdvLD status.*
(TIF)

Table S1 **List of ICD-9 CM diagnosis codes used to define HCV and AdvLD patients for both Medicare and non-Medicare patients.**
(DOCX)

Table S2 **2007 and 2009 HCV diagnosed and undiagnosed populations used in place of 2008 population values to test the sensitivity of the progression model outputs for 2015.**
(DOCX)

Table S3 **Total patient population growth rates by HCV status (2007–2009).**
(DOCX)
