# Supplementary figures and images for: Current and Future Disease Progression of the Chronic HCV Population in the United States
Source: PLoS One. 2013 May 21;8(5):e63959. doi: 10.1371/journal.pone.0063959 (PMC3660594; doi:10.1371/journal.pone.0063959)

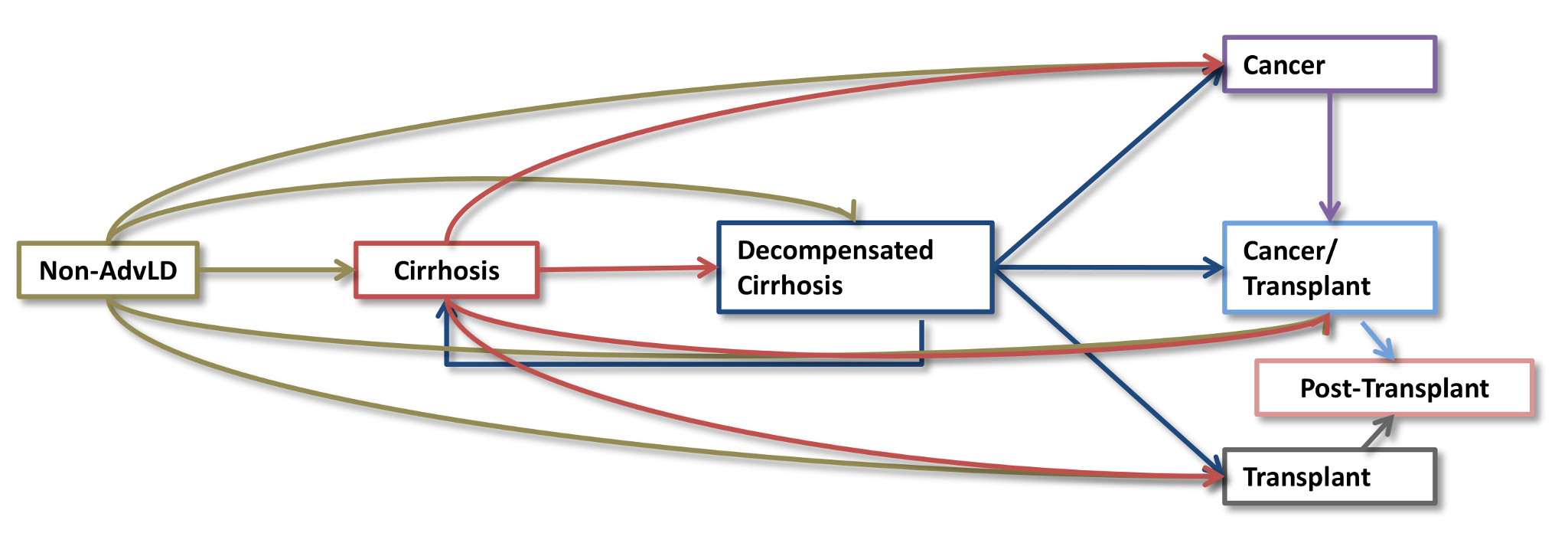

Supplement: Figure S1 — Rules for HCV patient disease progression. (TIF) [file pone.0063959.s001.tif]

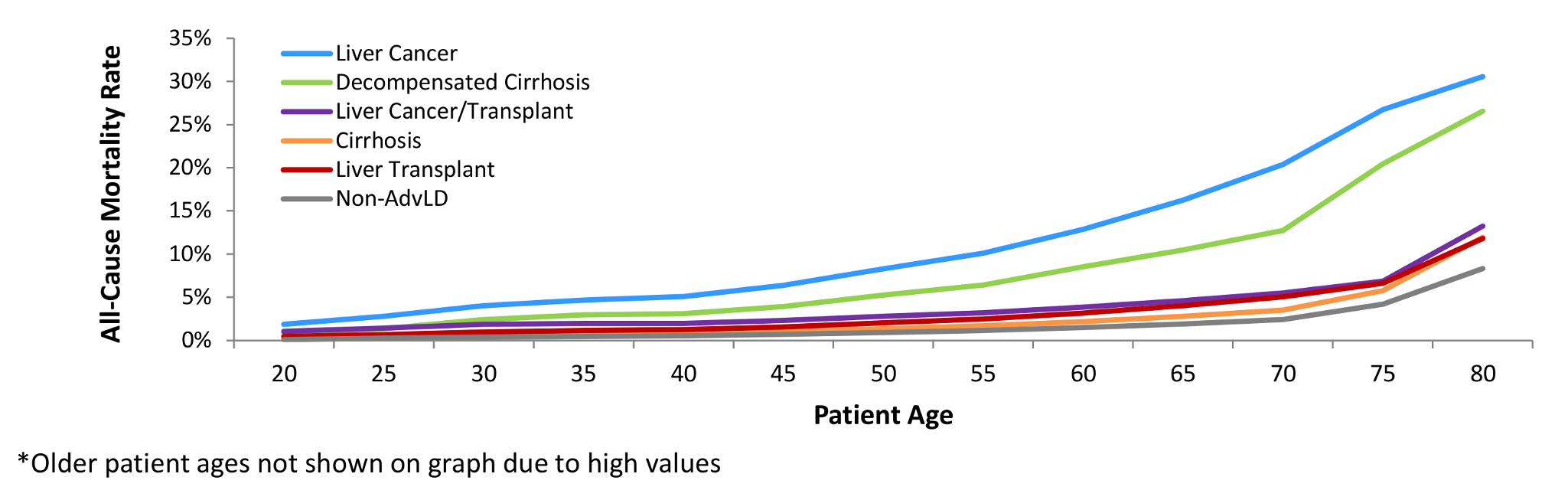

Supplement: Figure S2 — All-cause mortality rates by HCV-infected patient disease status*. (TIF) [file pone.0063959.s002.tif]

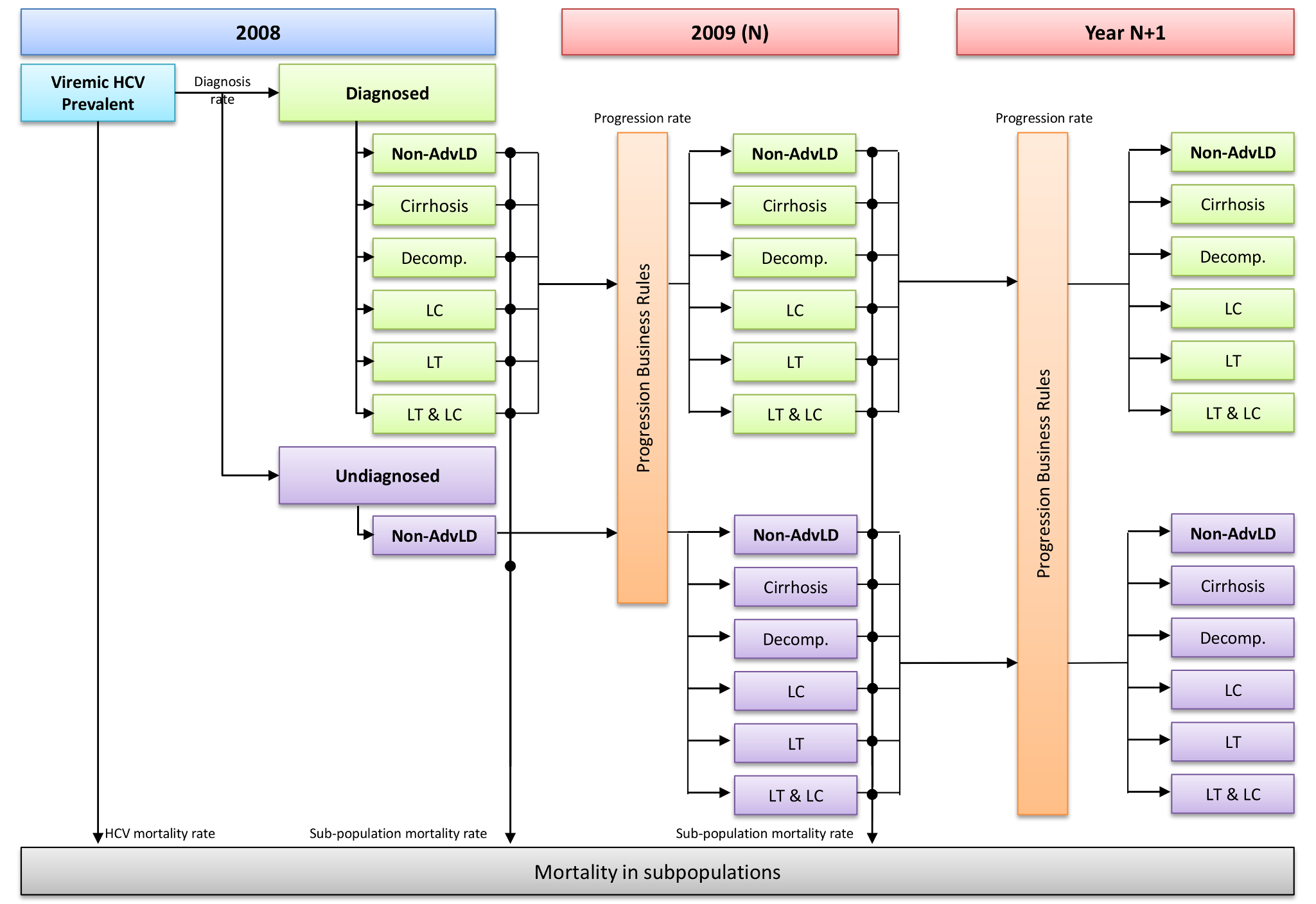

Supplement: Figure S3 — Progression model flow. (TIF) [file pone.0063959.s003.tif]

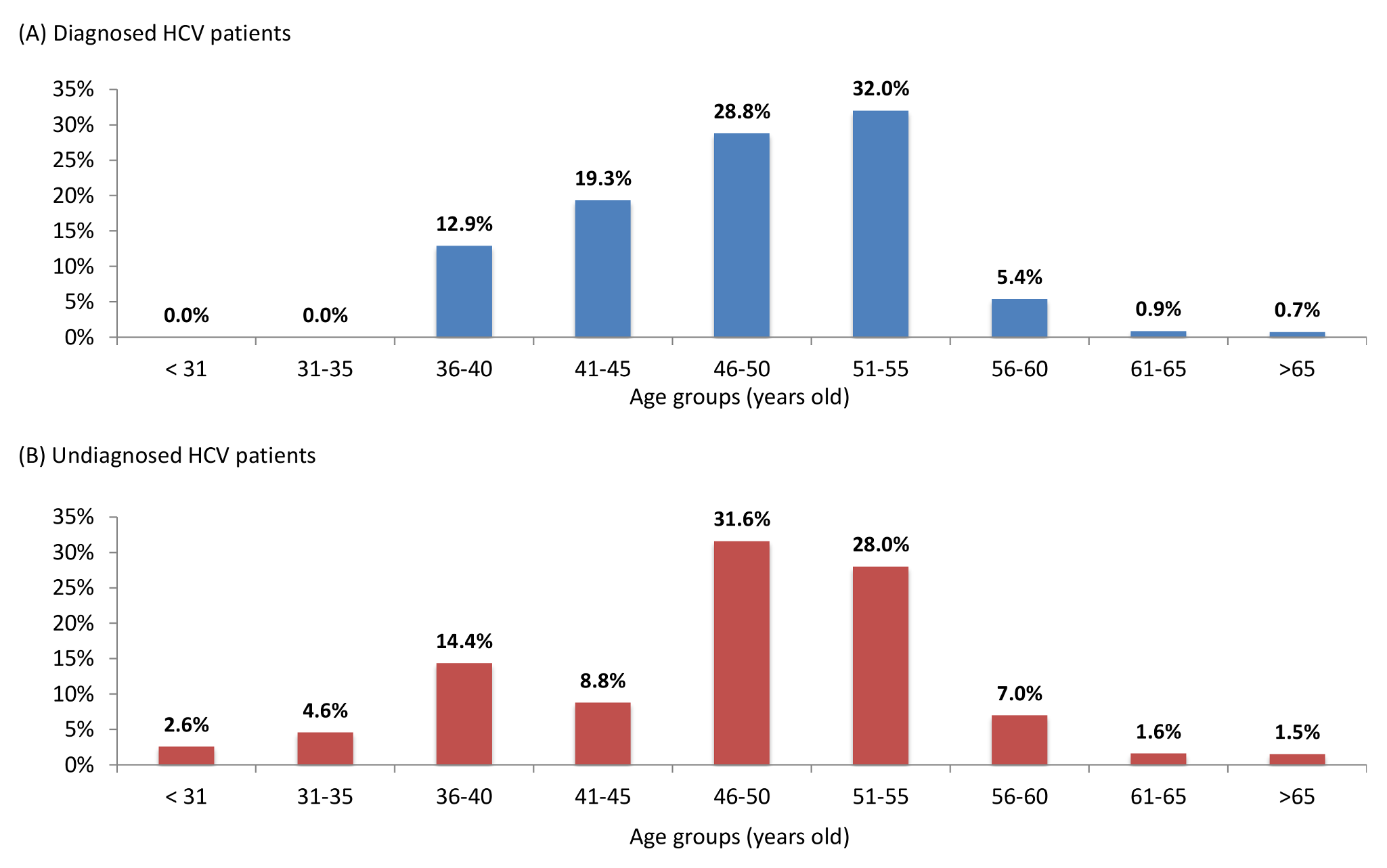

Supplement: Figure S4 — Age distribution of diagnosed and undiagnosed HCV-infected patients using 2005–06, 2007–08 NHANES data. (TIF) [file pone.0063959.s004.tif]

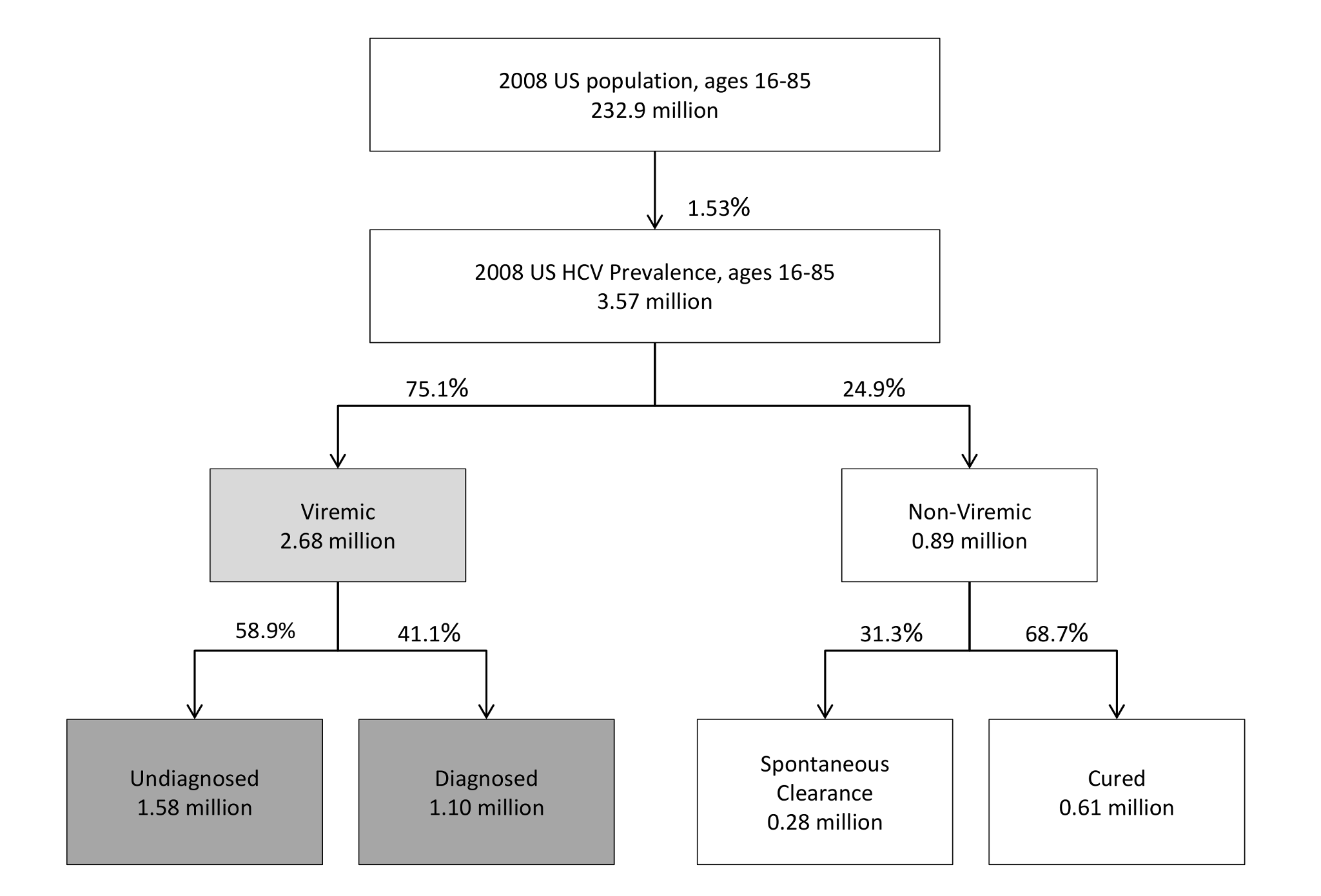

Supplement: Figure S5 — Prevalence of HCV in the US in 2008 by infection type (viremic/non-viremic). The viremic (i.e. chronic) population is further divided by diagnosis status. (TIF) [file pone.0063959.s005.tif]

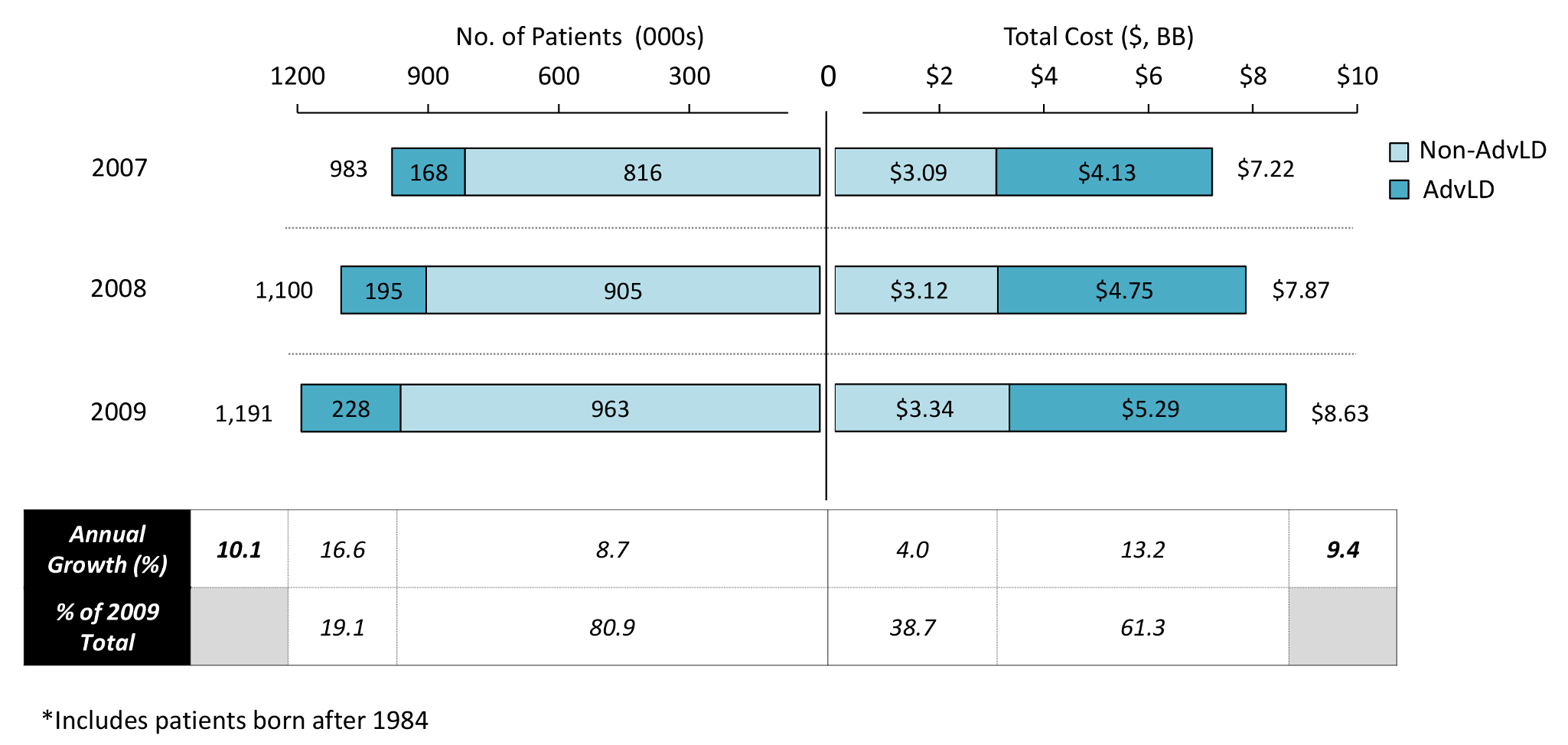

Supplement: Figure S6 — Total US diagnosed chronic HCV population (2007–2009): total patient count and total costs by AdvLD status.* (TIF) [file pone.0063959.s006.tif]
