# Supplementary material for: Current and Future Disease Progression of the Chronic HCV Population in the United States
Source: PLoS One. 2013 May 21;8(5):e63959. doi: 10.1371/journal.pone.0063959 (PMC3660594; doi:10.1371/journal.pone.0063959)
Supplement: Table S1 — List of ICD-9 CM diagnosis codes used to define HCV and AdvLD patients for both Medicare and non-Medicare patients. (DOCX) [file pone.0063959.s007.docx]

Table S1

| HCV ICD-9 Diagnosis Codes of Interest | | | |
| --- | --- | --- | --- |
| ICD-9 Code | Description | Code Type | Disease |
| 070.41 | ACUTE HEPATITIS C WITH HEPATIC | Diagnosis | HCV |
| 070.44 | CHRONIC HEPATITIS C W/HEPATIC | Diagnosis |  |
| 070.51 | ACUTE HEP C W/O MENTION HEP CO | Diagnosis |  |
| 070.54 | CHRONIC HEP C W/O MENTION HEP | Diagnosis |  |
| 070.70 | UNS VIRAL HEPATITIS C W/O HEP | Diagnosis |  |
| 070.71 | UNS VIRAL HEPATITIS C W/HEP CO | Diagnosis |  |
| V02.62 | HEPATITIS C CARRIER | Diagnosis |  |
| 571.2 | ALCOHOLIC CIRRHOSIS OF LIVER | Diagnosis | Cirrhosis |
| 571.5 | CIRRHOSIS LIVER W/O MENTION AL | Diagnosis |  |
| 571.6 | BILIARY CIRRHOSIS | Diagnosis |  |
| 070.44 | CHRONIC HEPATITIS C W/HEPATIC | Diagnosis | Decompensated Cirrhosis |
| 070.71 | UNS VIRAL HEPATITIS C W/HEP CO | Diagnosis |  |
| 273.2 | OTHER PARAPROTEINEMIAS | Diagnosis |  |
| 348.3 | UNSPECIFIED ENCEPHALOPATHY | Diagnosis |  |
| 348.30 | ENCEPHALOPATHY, UNSPECIFIED | Diagnosis |  |
| 348.31 | METABOLIC ENCEPHALOPATHY | Diagnosis |  |
| 348.39 | OTHER ENCEPHALOPATHY | Diagnosis |  |
| 456.0 | ESOPHAGEAL VARICES WITH BLEEDI | Diagnosis |  |
| 456.1 | ESOPH VARICES WITHOUT MENTION | Diagnosis |  |
| 456.2 | ESOPH VARICES IN OTH DIS | Diagnosis |  |
| 456.20 | ESOPH VARICES W/BLEED DZ CLASS | Diagnosis |  |
| 456.21 | ESOPH VARIC W/O BLEED DZ CLASS | Diagnosis |  |
| 571.4 | CHRONIC HEPATITIS | Diagnosis |  |
| 572.2 | HEPATIC COMA | Diagnosis |  |
| 572.3 | PORTAL HYPERTENSION | Diagnosis |  |
| 572.4 | HEPATORENAL SYNDROME | Diagnosis |  |
| 582.2 | CHRON GLN W/LES MEMBRANOPROLIF | Diagnosis |  |
| 583.2 | NEPHRIT&NEPHROP-LES MEMBRNPROL | Diagnosis |  |
| 782.4 | JAUNDICE UNSPECIFIED NOT OF NE | Diagnosis |  |
| 789.5 | ASCITES | Diagnosis |  |
| 789.59 | SYMPTOM, OTHER ASCITES | Diagnosis |  |
| 585.6 | RENAL DISEASE, END STAGE | Diagnosis | ESRD |
| 155 | MALIGNANT NEOPLASM LIVER | Diagnosis | Liver Cancer |
| 50.5 | LIVER TRANSPLANT | Procedure | Liver Transplant |
| 50.51 | AUXILIARY LIVER TRANSPLANT | Procedure |  |
| 50.59 | OTHER TRANSPLANT OF LIVER | Procedure |  |
| V42.7 | LIVER REPLACED BY TRANSPLANT | Diagnosis |  |
