# Supplementary material for: Current and Future Disease Progression of the Chronic HCV Population in the United States
Source: PLoS One. 2013 May 21;8(5):e63959. doi: 10.1371/journal.pone.0063959 (PMC3660594; doi:10.1371/journal.pone.0063959)
Supplement: Table S2 — 2007 and 2009 HCV diagnosed and undiagnosed populations used in place of 2008 population values to test the sensitivity of the progression model outputs for 2015. (DOCX) [file pone.0063959.s008.docx]

Table S2

| **HCV Status** | **2008 Population** | **2007 Population** | **2009 Population** |
| --- | --- | --- | --- |
| **Diagnosed**  *Non-AdvLD*  *AdvLD* | 1,100,000  *905,000*  *195,000* | 983,000  *816,000*  *167,000* | 1,191,000  *963,000*  *228,000* |
| **Undiagnosed Patients** | 1,576,000 | 1,700,000 | 1,479,000 |
| **Total Population** | **2,676,000** | **2,683,000** | **2,700,000** |
