# Supplementary material for: Current and Future Disease Progression of the Chronic HCV Population in the United States
Source: PLoS One. 2013 May 21;8(5):e63959. doi: 10.1371/journal.pone.0063959 (PMC3660594; doi:10.1371/journal.pone.0063959)
Supplement: Table S3 — Total patient population growth rates by HCV status (2007–2009). (DOCX) [file pone.0063959.s009.docx]

Table S3

| **Birth Cohort** | **HCV Status** | **Annual Growth (2007-2009)** |
| --- | --- | --- |
| 1910 - 1929 | **All Patients** | -15.0% |
|  | Non-AdvLD | -14.4% |
|  | All AdvLD | -17.9% |
|  | Cirrhosis | -21.8% |
|  | Decompensated Cirrhosis | -18.4% |
|  | Liver Cancer | -10.2% |
|  | Liver Transplant | -7.8% |
|  | Liver Transplant & Liver Cancer | -34.5% |
| 1930 - 1934 | **All Patients** | -9.3% |
|  | Non-AdvLD | -9.8% |
|  | All AdvLD | -7.3% |
|  | Cirrhosis | -14.2% |
|  | Decompensated Cirrhosis | -5.3% |
|  | Liver Cancer | -1.9% |
|  | Liver Transplant | -2.2% |
|  | Liver Transplant & Liver Cancer | -23.9% |
| 1935 - 1939 | **All Patients** | -5.0% |
|  | Non-AdvLD | -4.9% |
|  | All AdvLD | -5.6% |
|  | Cirrhosis | -7.1% |
|  | Decompensated Cirrhosis | -6.4% |
|  | Liver Cancer | 1.5% |
|  | Liver Transplant | -4.5% |
|  | Liver Transplant & Liver Cancer | -19.3% |
| 1940 - 1944 | **All Patients** | 6.8% |
|  | Non-AdvLD | 6.7% |
|  | All AdvLD | 7.6% |
|  | Cirrhosis | 4.9% |
|  | Decompensated Cirrhosis | 7.9% |
|  | Liver Cancer | 17.9% |
|  | Liver Transplant | -4.5% |
|  | Liver Transplant & Liver Cancer | 0.4% |
| 1945 - 1949 | **All Patients** | 8.7% |
|  | Non-AdvLD | 5.4% |
|  | All AdvLD | 20.3% |
|  | Cirrhosis | 17.8% |
|  | Decompensated Cirrhosis | 20.1% |
|  | Liver Cancer | 37.1% |
|  | Liver Transplant | 14.4% |
|  | Liver Transplant & Liver Cancer | 25.1% |
| 1950 - 1954 | **All Patients** | 9.4% |
|  | Non-AdvLD | 8.0% |
|  | All AdvLD | 14.5% |
|  | Cirrhosis | 11.8% |
|  | Decompensated Cirrhosis | 14.3% |
|  | Liver Cancer | 35.5% |
|  | Liver Transplant | 10.8% |
|  | Liver Transplant & Liver Cancer | 21.0% |
| 1955 - 1959 | **All Patients** | 11.3% |
|  | Non-AdvLD | 8.9% |
|  | All AdvLD | 22.9% |
|  | Cirrhosis | 19.1% |
|  | Decompensated Cirrhosis | 24.9% |
|  | Liver Cancer | 38.8% |
|  | Liver Transplant | 15.2% |
|  | Liver Transplant & Liver Cancer | 42.0% |
| 1960 - 1964 | **All Patients** | 12.4% |
|  | Non-AdvLD | 11.3% |
|  | All AdvLD | 18.0% |
|  | Cirrhosis | 9.2% |
|  | Decompensated Cirrhosis | 24.5% |
|  | Liver Cancer | 45.1% |
|  | Liver Transplant | 16.2% |
|  | Liver Transplant & Liver Cancer | 27.3% |
| 1965 - 1969 | **All Patients** | 12.9% |
|  | Non-AdvLD | 12.2% |
|  | All AdvLD | 18.1% |
|  | Cirrhosis | 12.2% |
|  | Decompensated Cirrhosis | 19.9% |
|  | Liver Cancer | 23.2% |
|  | Liver Transplant | 35.7% |
|  | Liver Transplant & Liver Cancer | 36.9% |
| 1970 - 1974 | **All Patients** | 16.4% |
|  | Non-AdvLD | 15.5% |
|  | All AdvLD | 26.2% |
|  | Cirrhosis | 37.8% |
|  | Decompensated Cirrhosis | 16.1% |
|  | Liver Cancer | 34.1% |
|  | Liver Transplant | 203.2% |
|  | Liver Transplant & Liver Cancer | 41.4% |
| 1975 - 1979 | **All Patients** | 15.8% |
|  | Non-AdvLD | 15.7% |
|  | All AdvLD | 17.3% |
|  | Cirrhosis | 8.9% |
|  | Decompensated Cirrhosis | 27.5% |
|  | Liver Cancer | 39.6% |
|  | Liver Transplant | -63.7% |
|  | Liver Transplant & Liver Cancer | N/D |
| 1980 - 1984 | **All Patients** | 26.3% |
|  | Non-AdvLD | 25.8% |
|  | All AdvLD | 34.2% |
|  | Cirrhosis | 26.5% |
|  | Decompensated Cirrhosis | 50.0% |
|  | Liver Cancer | 42.4% |
|  | Liver Transplant | -62.4% |
|  | Liver Transplant & Liver Cancer | -2.1% |
| 1985 - 2009 | **All Patients** | 16.9% |
|  | Non-AdvLD | 16.9% |
|  | All AdvLD | 16.3% |
|  | Cirrhosis | 44.7% |
|  | Decompensated Cirrhosis | 14.0% |
|  | Liver Cancer | 0.0% |
|  | Liver Transplant | -23.2% |
|  | Liver Transplant & Liver Cancer | -100.0% |
